# Host-parasite relationships in the genome

**DOI:** 10.1186/1741-7007-9-67

**Published:** 2011-10-10

**Authors:** John FY Brookfield

**Affiliations:** 1 School of Biology, University of Nottingham, University Park, Nottingham, NG7 2RD, UK

## Abstract

Transposable elements are best interpreted as genomic parasites, proliferating in genomes through their over-replication relative to the rest of the genome. A new study examining correlations across *Drosophila *species between transposable element numbers and rates of host evolution has brought into focus one of the most complex questions in transposable element biology-what it is that determines the proportion of the genome that is transposable elements.

See research article: http://www.biomedcentral.com/1471-2148/11/258/

## Commentary

Evolutionary biologists are ambitious people. They seek to explain why organisms are the way that they are, and to do this through knowledge of the environments to which organisms have adapted through the evolutionary process. If we wish to ask if organisms are optimally adapted to their environment, we need to infer, from the environment, what optimal phenotypic adaptation would look like. Then, if the inferred optimal phenotypes are seen, this explains why organisms are the way they are.

This approach plays a large role in evolutionary biology, and its successes have been documented. But there are many reasons, such as the absence of appropriate genetic variation in an adapting lineage, or a rapidly changing abiotic environment, why the approach will often fail. Most obviously, it will fail when the environment that is being adapted to consists of a competing, or predator, or prey, or host or parasite lineage that is itself undergoing a process of counter-adaptation. For host-parasite interactions, for example, a parasite showing the optimal phenotype will show adaptation to a particular phenotype displayed by its host. The specificity of the adaptation makes it likely that a better adapted host could exist, one able to escape from this parasite's harmful effects. If so, the current host necessarily has a suboptimal phenotype. Indeed, cycles of co-evolutionary change are a general feature of models that study expected changes in allele frequencies in host and parasite populations. In such models, a snapshot of host and parasite phenotypes at a given evolutionary time will show at most only one and, probably, neither of the lineages to be optimally adapted to the environment defined by the other.

Within the genome there are parasites in the form of transposable elements (TEs)-DNA sequences that can move to new genomic locations, either by copying themselves or by excision and re-insertion- and their hosts comprise the rest of the genome, on which selection operates on the basis of the survival and reproduction of individuals. For selfish genetic elements such as TEs [1] their ability to spread in the absence of selection at the level of the host allows them to persist even if their net effects on host fitness are negative.

TEs can, of course, sometimes be beneficial to their hosts, by creating insertion mutations that assist in the host's process of adaptive evolution [2]. Given their abundance and their capacity to act as mutagens, it would be strange indeed if insertion of TEs never created adaptive changes. Equally, long after their insertion and fixation in the genome, their DNAs can mutate and can sometimes create functions, particularly in controlling expression of adjacent genes, that are useful to their hosts [3].

If we wish to interpret the observations of the particular host-parasite interaction that TEs and their genomic context constitutes, we thus must bear in mind the inevitable inadequacy of simply assuming optimal adaptation in the two participants in the interaction. Rather, we need to attempt to trace the historical process of adaptation and counter-adaptation in the two lineages, since the phenotype of one lineage may represent an adaptation to a phenotypic trait in the other lineage that that lineage no longer possesses. We must remember that the TEs that we see are those persisting today, so there is biased ascertainment-we only observe interactions where the parasites are 'winning' in that sense. Also, the co-evolutionary nature of host-parasite interactions warns us not to assume that TE-host interactions will lead to the stable equilibria that population geneticists find so useful in estimating evolutionary parameters [4].

In addition to the parasite's interaction with the host, there are interactions between TEs of different families and classes, in a kind of community ecology [5]. In particular, asking what determines a TE family's copy number is, in some ways, analogous to asking what determines the relative abundance of different species in ecological communities, a question that, regrettably, ecology has not been very successful in answering.

How can we approach the question of copy number? The genomic abundance of a family of TEs represents the integral of its birth and death processes since it invaded the genome. Castillo *et al*. [6] consider both processes. The death of elements happens randomly by stochastic loss through genetic drift, but also through selection at the level of the host. The loss of element copies through selection will depend on the strength of selection against insertions of the elements. There will be enormous variability between sites in their selection strength, but it may be that some sites are weakly deleterious, effectively neutral in small populations, but selected in large populations. For weakly deleterious sites, fixation of the element insertion by drift may occur, thus preventing selective loss, and, in this way, the selective removal of weakly harmful elements will typically be attenuated in small populations. Castillo *et al*. [6] argue that if amino acid changes to proteins are also sometimes weakly deleterious, the rate of amino acid change will be elevated in small populations for the same reason. Thus, one might expect that there will be a positive correlation between the rate of change in the amino acids, measured by a dN/dS ratio (the ratio of the rate of change in the amino acid sequence of proteins to the rate of change in synonymous sites in the genes encoding them), and the transposable element abundance, since both will reflect an underlying variation in the effective population size over recent evolutionary time.

Equally, the abundance of elements will depend on their birth process. One factor that acts to counter TE spread is the PIWI-interacting system of interfering RNAs (piRNAs). These are short RNAs, 20 to 30 bases in length in *Drosophila*, and typically derived from either the sense or the anti-sense strands of TEs [7]. A complex machinery of proteins controls the piRNAs and it is possible to show (by studies of expression of TE RNAs in flies mutant for proteins in the pathway [8]) that the proteins' wild-type function is to lower TE expression, and, with it, transposition.

For these proteins, Castillo *et al*. [6] supply a different prediction, that these will be involved in a co-evolutionary 'arms race' with the TEs, and the rate of adaptive evolution in the proteins will be highest in genomes with the most TEs. This is predicted because it is in these genomes that the TEs are selecting the most strongly for adaptive evolution in proteins of the piRNA machinery. These two predictions were tested using the 12 sequenced *Drosophila *genomes [6], and investigating the dN/dS ratio as a measure of the types of selection (purifying and adaptive) that have occurred in the evolutionary changes connecting these diverse species.

The results of the study were perhaps surprising. The correlation between TE copy number and the dN/dS ratio genome-wide was, in fact, negative. The positive correlation expected relies, of course, on some TE insertions falling in the narrow window of selection coefficients such that they would be effectively neutral in some (smaller) *Drosophila *populations but effectively selectively eliminated in larger populations. In fact [6], in *Drosophila *there seems no general correlation between high TE numbers and small population sizes. Perhaps large populations are invaded by more TE families because they are more geographically widespread and, as a result, are more prone to horizontal transfer of new elements.

In addition, while it was expected that there would be a positive correlation between the piRNA machinery proteins' dN/dS ratios and the TE abundance, these proteins' dN/dS correlation with TE abundance was, on average, even more negative than that of a control set of proteins.

These results illustrate the logical difficulties of interpreting host-parasite interactions without considering an explicit time dimension. Note that there is a subtle difference between the roles of time in the two theoretical predictions. For the comparison between genomic dN/dS and TE abundance, a positive correlation was expected because of the similarity between the process creating high dN/dS and the process leading to high TE abundance. For the comparison between the piRNA machinery genes' dN/dS and TE abundance, the positive correlation was expected because having had high TE abundance in the past would have created high dN/dS in the genes. So the second hypothesis looks at the time course of evolutionary change in a subtly different way from the first since here the dN/dS observed should be positively correlated with TE abundance in the past, not with TE abundance in the present.

For the piRNA machinery genes, as the authors point out [6], the model can be wrong in two different ways. It could be that high transposable element numbers did increase selection on the genes, but this consisted of greater purifying selection, through which amino acid changes reducing the proteins' function were more efficiently eliminated, and which will lower dN/dS. Indeed, there is evidence for enhanced codon usage bias when TE abundance is high, which will have a consequence for accuracy as well as speed of translation. But, reversing the causality, one can also argue that rapid evolutionary change in the genes has been successful in creating a more effective anti-TE mechanism, therefore driving down the TE numbers. It was the TE abundance in the past that drove the piRNA genes' evolution, and, as a result, in fly lineages where TE numbers were once high, they may now be unusually low. In this classic host-parasite interaction, TE numbers in the present may be a poor indicator of TE numbers in the past.

The message, it seems, is that trying to identify complex host-parasite co-evolutionary dynamics by examination of single time points will be as difficult in genomic studies as it is elsewhere. But the capacity of TEs to 'die' as active elements but to live on in the genome as 'molecular fossils' will give TE biologists a tool not available to other students of host-parasite interactions.

## References

[B1] Werren JH Selfish genetic elements, genetic conflict and evolutionary innovation Proc Natl Acad Sci USA 2011 108 10863 10870 10.1073/pnas.1102343108 21690392 PMC3131821

[B2] González J Lenkov K Lipatov M MacPherson M Petrov DA High rate of recent transposable element-induced adaptation in *Drosophila melanogaster* PLoS Biol 2008 6 e251 10.1371/journal.pbio.0060251 18942889 PMC2570423

[B3] Lowe CB Bejerano G Haussler D Thousands of human mobile element fragments undergo strong purifying selection near developmental genes Proc Natl Acad Sci USA 2007 104 8005 8010 10.1073/pnas.0611223104 17463089 PMC1876562

[B4] Le Rouzic A Boutin TS Capy P Long-term evolution of transposable elements Proc Natl Acad Sci USA 2007 104 19375 19380 10.1073/pnas.0705238104 18040048 PMC2148297

[B5] Venner S Feschotte C Biemont C Dynamics of transposable elements: towards a community ecology of the genome Trends Genet 2009 25 317 323 10.1016/j.tig.2009.05.003 19540613 PMC2945704

[B6] Castillo DM Mell JC Box KS Blumenstiel JP Molecular evolution under increasing transposable element burden in *Drosophila*: A speed limit on the evolutionary arms race BMC Evol Biol 2011 11 258 10.1186/1471-2148-11-258 21917173 PMC3185285

[B7] Brennecke J Aravin AA Stark A Dus M Kellis M Sachidanandam R Hannon GJ Discrete small RNA-generating loci as master regulators of transposon activity in *Drosophila* Cell 2007 128 1089 1103 10.1016/j.cell.2007.01.043 17346786

[B8] Lu J Clark AG Population dynamics of PIWI-interacting RNAs (piRNAs) and their targets in *Drosophila* Genome Res 2010 20 212 227 10.1101/gr.095406.109 19948818 PMC2813477

